# Supplementary material for: NMDA-receptor-dependent plasticity in the bed nucleus of the stria terminalis triggers long-term anxiolysis
Source: Nat Commun. 2017 Feb 20;8:14456. doi: 10.1038/ncomms14456 (PMC5321732; doi:10.1038/ncomms14456)
Supplement: Supplementary Information — Supplementary Figures. [file ncomms14456-s1.pdf]

Supplementary Figure 1

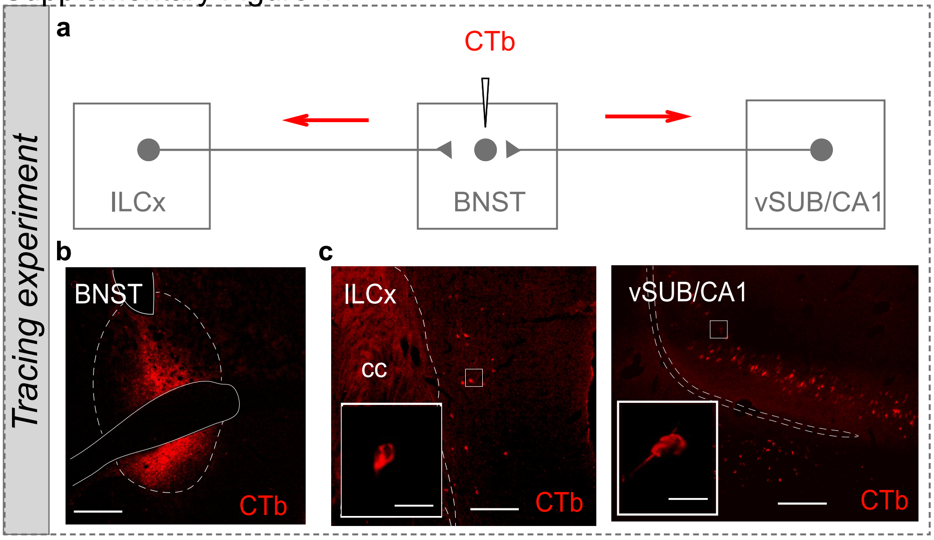

Supplementary Figure 1: The amBNST receives strong innervations from both ILCx and vSUB/CA1. a. Experimental protocol. b. Injection site of retrograde tracer CTb in the amBNST. Scale bar: 0.5 mm. c. Detection of the retrograde tracer CTb in the vSUB/CA1 and in the ILCx. Scale bar: 0.1 mm. inset: scale bar = 10 µm.

Supplementary Figure 2

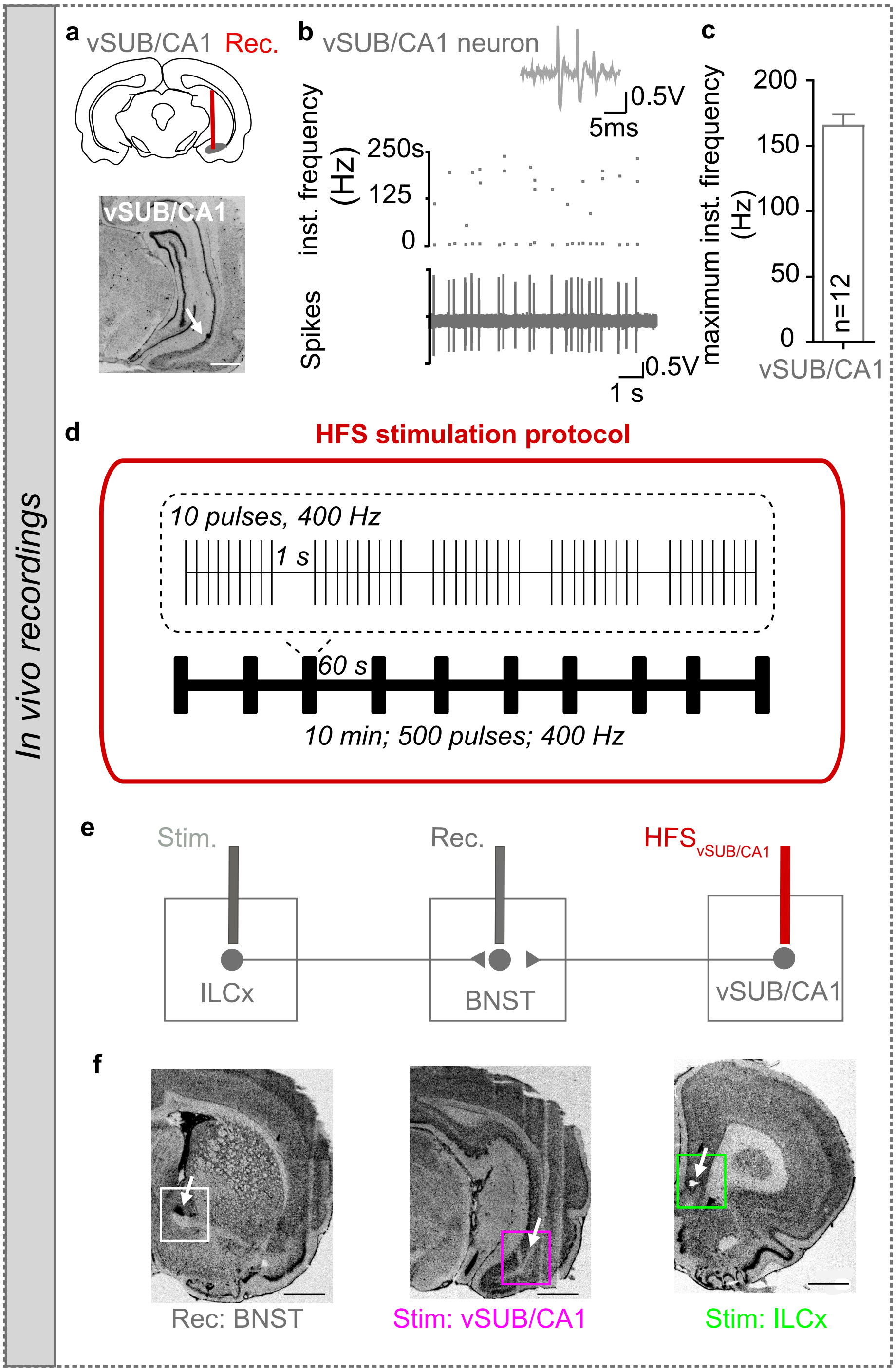

Supplementary Figure 2: High frequency stimulation protocol. a. Experimental protocol (top) and example of histological of recording area (bottom). Scale: 0.5 mm. b. Example trace of a vSUB/CA1 neuron recorded with the instantaneous frequency (inst. Frequency) and an inset , scale bar 5 ms. c. Histogram of the mean average of the maximum instantaneous frequency of vSUB/CA1 neurons expressed in Hz. d. High frequency stimulation protocol. e-f. Experimental protocol and histological control of recording and stimulating areas where $HFS_{vSUB/CA1}$ has been assessed. Scale bar: 0.5 mm.

Supplementary Figure 3

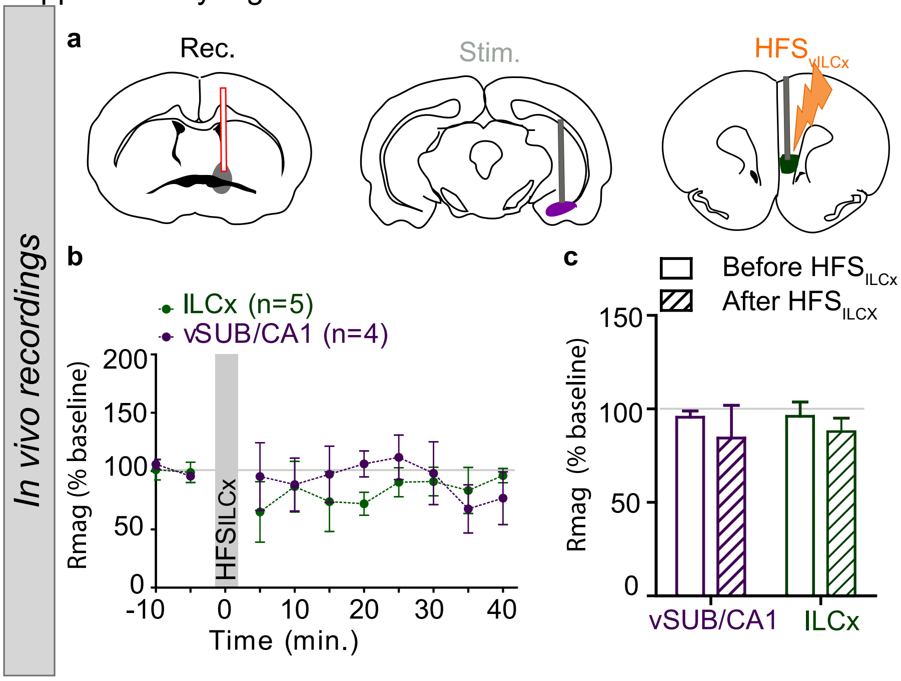

Supplementary Figure 3: HFS applied in the ILCx do not induce plasticity in the ILCx nor in the vSUB. a. Experimental protocol. b. Kinetic (left) and quantification (right) of the mean percentage change (± sem) in ILCx evoked spike probability (green) and in vSUB/CA1 evoked spike probability (purple), normalized to the baseline, after HFS_{ILCx}. Rmag, excitatory response magnitude.

Supplementary Figure 4

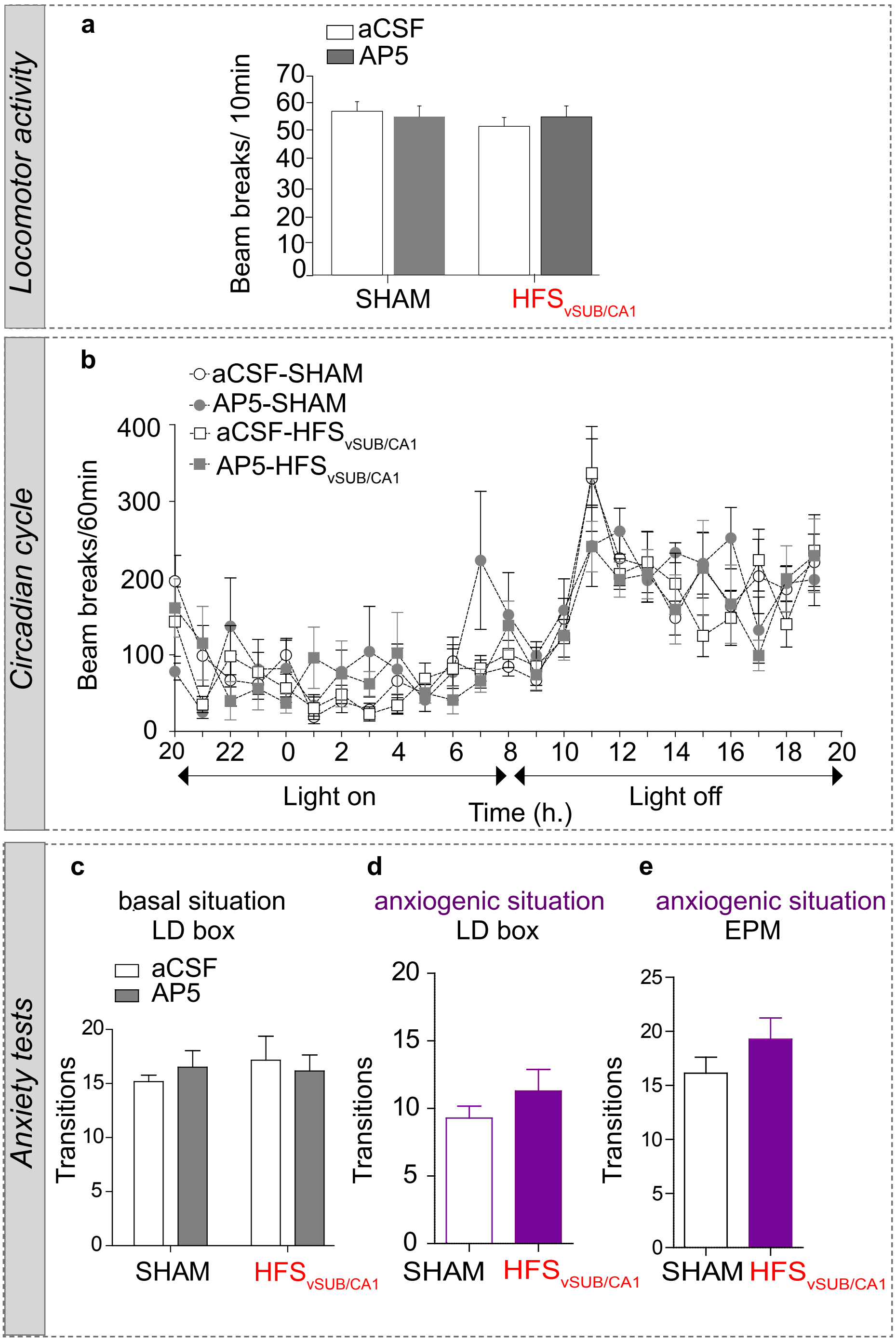

Supplementary Figure 4: Locomotor activity and circadian rhythms of general activity in sham or $HFS_{vSUB/CA1}$ treated animals after ACSF or AP5 infusion in the amBNST. a. Locomotor activity in a novel environment. b. circadian rhythms of general activity. No between-group differences in novelty-induced locomotor activity or circadian rhythms of general activity excluded differences unrelated to anxiety. c. Histograms showing the number of transitions in the light-dark box test after AP5 (grey) or vehicle in the BNST (aCSF, white) followed by $HFS_{vSUB/CA1}$ or sham manipulation in basal situation. d-e. Histograms showing the number of transitions in the light-dark box test (d) and in the open arms in the EPM (e) in the SHAM group and in HFS $_{vSUB/CA1}$ group in anxiogenic situation.

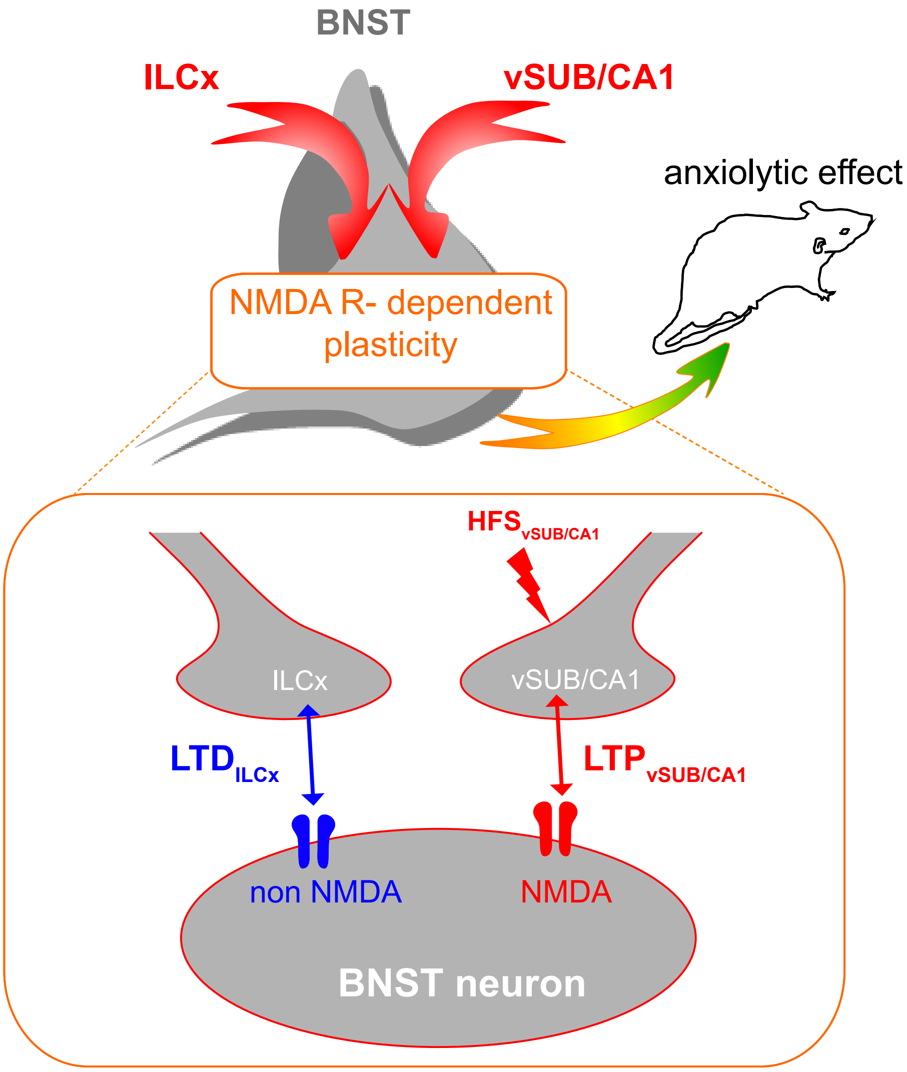

Supplementary Figure 5: NMDA-R-dependent plasticity in the amBNST induced by $HFS_{vSUB/CA1}$ triggers anxiolytic effect.

A proposed diagram of the circuit's mechanisms in response to $HFS_{vSUB/CA1}$. amBNST neurons integrate, at the single-cell level, inputs from both ILCx and vSUB/CA1. $HFS_{vSUB/CA1}$ induced a NMDA dependent LTP $_{vSUB/CA1}$ whereas it promoted an NMDA independent LTD_{ILCx}. In this model, *in vivo* NMDA-R dependent plasticity in amBNST neurons after $HFS_{vSUB/CA1}$ induced anxiolytic effect in rats.
